# A Good Example

**Published:** 1891-04-18

**Authors:** 


### Passing Topics.

### A GOOD EXAMPLE.

That the Midland Railway Company should have subscribed five thousand pounds to the fund for rebuilding the Derby Infirmary is a proof that a board of directors after all may possess bowels of compassion. It seems to testify, also, to the fact that a genius for originality still continues to haunt this great organization, and that the company which was the earliest to eliminate that concession to an old-fashioned threadbare gentility—the second-class carriage—which cut down the fares of the first class to democratic figures, and boldly bestowed upon the third the advantage of travelling at express speed, is actuated by deeper feelings than mere commercial acuteness, and is prepared to demonstrate that none need blush to be philanthropic, when a great public company, with a legion of shareholders keen for dividends, makes free to be generous.

It is a somewhat remarkable circumstance that this, and, so far as we know, the only example of substantial help being given to a voluntary institution by any one of the great railway companies running into London, should have emanated from the company whose headquarters are situated away from the metropolis—that is, in Derby itself. Here we have, apparently, another illustration of the advantage to a charity of localisation. A Metropolitan hospital would have appealed in vain. It was but the other day that one of the Secretaries, in giving evidence before the Lords' Committee, put forth a plaintive plea for some recognition of indebtedness by the several large railway companies whose lines converge upon the neighbourhood of his hospital, and whose servants make large demands upon it for attention. The suggestion did not appear to meet with encouragement at the hands of the Committee. Yet it looks eminently reasonable. We might ask, What would be the position of the companies [and of other large employers of labour if there were no voluntary hospitals to which the victims of accidents could be sent? Is it not certain that the companies would be under the necessity of providing hospitals at their own cost? And if this be admitted, as we think it must be, it follows there is a distinct moral obligation to assist the institution which saves them a large outlay, unavoidable under other circumstances.

Again, it was calculated by the Secretary in question that a certain company, which, to its credit, gives an annual subscription of £50, for there is no possible way of compelling it to give anything, sent patients last year who cost the hospital nearly £400. Where did the balance of £350 come from? Clearly from the pockets of the subscribers pure and simple—that is subscribers who neither obtain nor look for a *quid pro quo*. One may fairly ask why these philanthropic individuals should assume a burden rightly belonging to a powerful company?

Unless we are much mistaken, even subscriptions of £50 are few and far between. It is not an unknown circumstance that a subscription should be refused upon the ground that "the company has no funds applicable to such a purpose," a formula the hospitals might often use with absolute truth when called on for aid, and the difficulties in the way of obtaining assistance from any joint stock undertaking are usually, we believe, enormous. All the more do we welcome and applaud the action of the Midland Railway directors, and we trust that the example may not be lost upon the other companies. When the time comes for a juster view of such matters to be taken, the enlightenment of that later period will look back with wonder upon our worship of the voluntary system of medical relief, with its excesses, anomalies, and short-comings. Agricola.

